# The metabolic syndrome and renal function in an African cohort infected with human immunodeficiency virus

**DOI:** 10.4102/sajhivmed.v19i1.813

**Published:** 2018-09-20

**Authors:** Edith Phalane, Carla M.T. Fourie, Aletta E. Schutte

**Affiliations:** 1 Hypertension in Africa Research Team, North-West University, South Africa; 2 Medical Research Council Unit for Hypertension and Cardiovascular Disease, Faculty of Health Sciences, North-West University, South Africa

## Abstract

**Introduction:**

The human immunodeficiency virus (HIV) is often accompanied by renal dysfunction. It is expected that metabolic syndrome (MetS) may exacerbate renal impairment.

**Objective:**

We therefore determined the prevalence of MetS and the association thereof with renal function in a South African cohort infected with HIV.

**Methods:**

We matched 114 HIV-infected (77.3% on antiretroviral therapy [ART] and 22.7% ART-naïve) and 114 HIV-uninfected individuals according to age, sex and locality. We examined cardiovascular, anthropometric and metabolic measurements and determined the MetS. Renal function was assessed using standardised procedures.

**Results:**

The prevalence of MetS was lower in the HIV-infected individuals as compared to the uninfected individuals (28% vs. 44%, *p* = 0.013). The HIV-infected group presented with a lower body mass index (BMI) and waist circumference (WC) (all *p* < 0.001), as well as blood pressure (BP) (*p* ≤ 0.0021). The results were confirmed when comparing the HIV-infected group using ART (*N* = 85) and the HIV-uninfected group. When comparing the HIV-infected individuals with MetS to the HIV-uninfected individuals with MetS, no differences in BP were seen. With regard to renal function, the HIV-infected individuals with MetS (*n* = 32) had 43% higher urinary albumin-creatinine ratio (uACR) compared to the HIV-uninfected individuals with MetS, after adjusting for age, sex and WC (*p* = 0.032). None of the other renal function markers differed after adjustments for WC or BMI.

**Conclusion:**

The HIV-infected Africans with MetS had almost twofold higher uACR, despite the low prevalence of MetS, compared to their uninfected counterparts. The combination of HIV and MetS seemed to increase the risk for renal impairment.

## Introduction

The global burden of the human immunodeficiency virus (HIV) continues to rise with approximately 38.8 million people being infected worldwide. Sub-Saharan Africa contributes 75.4% of new infections globally.^1^ In South Africa, the prevalence of HIV was estimated at 6.12 million for the year 2015.^2^ The introduction of antiretroviral therapy (ART) to HIV-infected individuals has significantly improved mortality and morbidity,^3^ and HIV infection has now become a manageable chronic disease.^4^ However, the beneficial effects of ART are often overshadowed by comorbidities such as abnormal fat distribution,^5^ hypertension^6^ and dyslipidaemia.^7,8^

These comorbidities form part of metabolic syndrome (MetS),^6,9^ a multifaceted syndrome defined by a constellation of several cardiovascular risk factors.^10,11^ Metabolic syndrome is commonly reported among people living with HIV infection, and the prevalence is commonly affected by the type of ART regimen use.^6,12^ MetS is also an independent risk factor for renal disease^13^, and it is not clear if it is the MetS per se or its individual components that are the cause of the observed renal impairment.^13,14^

Human immunodeficiency virus infection is independently associated with microalbuminuria among Black and White Americans.^15^ Furthermore, Okpa et al.^16^ reported the prevalence of microalbuminuria at 15% among newly diagnosed HIV-infected individuals in Nigeria. Microalbuminuria does not only reflect renal dysfunction but is also a marker of systemic endothelial damage,^17^ which is linked to an elevated risk of kidney damage, cardiovascular disease and mortality.^18^ Kidney disease contributes significantly to morbidity and mortality in HIV-infected individuals.^19^

Tenofovir disoproxil fumarate (TDF), part of the first-line ART regimen in South Africa since April 2010,^20^ is potentially nephrotoxic.^21^ The prevalence of tenofovir-associated nephrotoxicity is estimated at 2.4%, and the effect is considered to be mild and tolerable.^22,23,24,25^ Prolonged use of tenofovir affects kidney function more than any other non-nucleoside reverse transcriptase inhibitors.

Few studies report on the prevalence of MetS and the association thereof with renal function among the South African population living with HIV. As renal dysfunction is related to both the use of ART (tenofovir) and MetS, we hypothesise that the HIV-infected population using ART and suffering from MetS may be at particular risk for renal impairment. We reported the MetS prevalence in this cohort to be 21% when the individuals were newly diagnosed as being HIV-infected and ART-naïve in 2005.^26^ Therefore, the aim of this study is, first, to determine the prevalence of the MetS 10 years later and, second, to determine the association of MetS with renal function in a South African cohort infected with HIV.

## Methods

### Study design and population

The Prospective Urban and Rural Epidemiological (PURE) study is a multinational longitudinal study examining the changes in lifestyle and causes of chronic diseases, through periodic standardised data collection.^27^ The PURE study focuses on urban and rural areas in 17 different low- and middle-income countries, including South Africa. In the North-West Province of South Africa, the PURE study participants were randomly recruited door-to-door from two main sites: Potchefstroom (urban) and Ganyesa (rural), and they were followed for 10 years.

Black men and women older than 35 years were included in the PURE study. When we recruited, the HIV-infected individuals were unaware of their status and were newly identified as being HIV-infected. For this cross-sectional study, we matched data for 114 HIV-infected individuals with 114 HIV-uninfected participants according to age, sex and locality (urban and rural areas). Of the 114 HIV-infected participants, 27 had been infected with HIV for five years (24%) and 87 were infected for 10 years (76%). The participants (*n* = 228) had complete data sets for all MetS components.

### Questionnaires

Questionnaires were used to collect data on socio-economic and demographic information, current health status, medical and family history, medication, as well as tobacco and alcohol use.

### Anthropometry

Anthropometric measurements were performed according to standardised procedures, as prescribed by the guidelines of the International Society for the Advancement of Kinanthropometry (ISAK).^28^ We measured the height of the participants to the nearest 0.1 cm with a stadiometer (Leicester height measure, Seca, Birmingham, UK) and the weight of the participants to the nearest 0.1 kg on a portable electronic scale (Precision Health Scale, A & D Company, Japan). Body mass index (BMI) was calculated. Waist circumference (WC) was recorded to the nearest 0.1 cm with a steel tape (Lufkin, Cooper Tools, Apex NC, USA).

### Blood pressure measurement

The participants were allowed to rest for 10 minutes before blood pressure (BP) measurements were taken. Duplicate brachial BP measurements were taken in the sitting position at 5-min intervals, using the validated OMRON M6 device (Omron Healthcare, Kyoto, Japan). An appropriate cuff size was used, and it was placed on the right arm over the brachial artery, with the arm supported at heart level and in a relaxed position. Pulse pressure and mean arterial pressure were calculated. Duplicate central systolic blood pressure (cSBP) was measured with the Sphygmocor XCEL device (Atcor Medical Pty. Ltd., Sydney, Australia), with the participant in the supine position.

### Biological sample collection

Prior to measurements, the participants were asked to fast overnight for a period of eight hours. A registered nurse collected the venous blood using a winged infusion set. The serum and plasma were prepared according to standardised procedures and were stored at -80 °C until analysis. A spot urine sample was collected and stored at -80 °C until analysis.

### Biochemical analysis

We analysed the serum samples with the Cobas Integra 400 plus (Roche Basel, Switzerland), and glucose, total cholesterol (TC), triglycerides (TG), low density lipoprotein cholesterol (LDL-c), high-density lipoprotein cholesterol (HDL-c), γ-glutamyl transferase (GGT) and creatinine levels were determined. Serum C-reactive protein (CRP) levels were determined by means of a particle-enhanced turbidimetric assay. Glycosylated haemoglobin (HbA1c) was determined using the D-10 Haemoglobin testing system (Bio-Rad, #220-0101) by means of ion-exchange high-performance liquid chromatography.

We also analysed the urinary creatinine and albumin levels using the Cobas Integra 400 plus (Roche, Basel, Switzerland) by means of a kinetic colorimetric assay.

The inter- and intra-assay coefficients of variability for the biochemical analyses were as follows: glucose (1.8%; 2.1%), TC (0.2%; 1.9%), HDL-c (1.1%; 1.0%), LDL-c (1.5%; 1.9%), GGT (1.8%; 1.8%), CRP (1.3%; 3.5%), HbA1c (0.9%; 1.3%), creatinine (1.4%, 2.5%) and albumin (1.9%; 2.2%).

### Human immunodeficiency virus testing and counselling

Participants were counselled before and after HIV testing by a registered counsellor. The HIV status was determined from whole blood according to the protocols of the South African Department of Health, using the first response rapid HIV card test (Premier Medical Corporation Limited, Daman, India). In the case of a positive result, the test was confirmed with an Abon (Biopharm Corporation Limited Hanyzhou, China) rapid card test. For the CD4+ count analyses, finger-prick blood was collected and the CD4+ counts were determined at the research site using a point-of-care device, the PIMA^TM^ CD4+ (Alere, Jena, Germany). Of the 114 HIV-infected participants, 77.3% were using ART and were on the first-line ART regimen, namely the fixed dose combination compared to 22.7% that were ART-naïve. The fixed dose combination consisted of TDF, efavirenz (EFV) and emtricitabine (FTC) under different generic names but with the same active ingredients, namely, Tribuss, Atripla, Atroiza, Trivenz and Odimune.

### Metabolic syndrome definition

We defined MetS using the criteria of the International Diabetes Federation (IDF): individuals with WC > 94 cm for men and > 80 cm for women and any of the other two: BP > 130/85 mmHg, TG > 1.7 mmol/L, HDL-c < 1.03 mmol/L for men and < 1.29 mmol/L for women and glucose > 5.6 mmol/L.^29^

### Renal function

Creatinine clearance (mL/min) was calculated using the Cockcroft-Gault formula as follows: ([140-age] * [weight in kg] * [1.23 if male OR 1.04 if female])/(serum creatinine in µmol/L).^21^ For creatinine clearance (CrCl), a cut-off value of < 50 mL/min was chosen, as the South African Department of Health recommends the use of tenofovir to be discontinued below this cut-off point.^30^ We calculated the estimated glomerular filtration rate (eGFR) (mL/min/1.73 m^2^) using the Chronic Kidney Disease Epidemiology Collaboration (CKD-EPI) equations.^21^ Black ethnicity was not included in the formula.^31^ We used the cut-off value of eGFR < 90 mL/min/1.72 m^2^.^32^ Urinary albumin creatinine ratio (uACR) was calculated and a cut-off value of 3 mg/mmol – 30 mg/mmol, which defines microalbuminuria, was used.^21^

### Statistical analysis

We performed statistical analysis using Statistica version 13 (Stasoft Inc., Tulsa, OK). Descriptive statistics were done for all the normally distributed variables and were presented as means and standard deviations. Variables which were not normally distributed were log transformed and presented as geometric means and 5th and 95th percentiles. An independent *t*-test was used to compare the means of the groups, and the Chi-square test was used for proportions of the categorical variables. Analyses of covariates were used to compare HIV-infected and HIV-uninfected groups while adjusting for WC. We also compared uACR between the HIV-infected with and without MetS, and the HIV-uninfected with and without MetS, after adjusting for age, sex and WC. Bonferroni tests were used in post hoc comparisons between the groups. The adjusted least square means were used to plot a bar graph comparing the four groups mentioned. Finally, we performed multivariate adjusted regression analyses with renal markers (CrCl, eGFR and uACR) as dependent variables in the total group, and in the HIV-uninfected and HIV-infected groups to determine the contributions of the MetS components towards renal function in the different groups. The independent variables that were entered into the model included age, sex, cSBP, WC, HDL-c, TG, HbA1C, CRP, MetS, HIV status, CD4+ cell count and ART. The significance level was set at *p* ≤ 0.05.

## Ethical consideration

Both the PURE study and this substudy were approved by the Health Research Ethics Committee (HREC) of the North-West University in South Africa (approval number: 00016-10-A1 and NWU-00035-16-S1). The study protocols conformed to the principles of the Declaration of Helsinki. The research information was conveyed in the participants’ home language by trained African fieldworkers. The participants gave informed consent to take part in the study.

## Results

Characteristics of the HIV-infected and matched HIV-uninfected groups are presented in Table 1. As a result of matching age, sex and locality, the groups were similar. The HIV-infected group presented with lower prevalence of MetS than the HIV-uninfected group (28% vs. 44%, *p* = 0.013). In those on ART (*N* = 85), the prevalence of MetS was 24.2%.

**TABLE 1:** Characteristics of the HIV-uninfected and HIV-infected individuals (2015).

Variable	HIV-uninfected *N* = 114	HIV-infected *N* = 114	*P*
Men, *N* (%)	23 (20.2)	23 (20.2)	-
Age, years	53.3 ± 5.5	53.4 ± 5.6	0.874
Urban *N* (%)	46 (40.4)	46 (40.4)	-
**Anthropometry**			
WC, cm	91.6 ( 70.5; 122.6)	81.7 ( 64.6; 109.1)	< 0.001
BMI, kg/m^2^	27.4 (18.0; 44.5)	22.8 (16.1; 34.5)	< 0.001
**Cardiovascular measurements**			
SBP, mmHg	133 ± 21	126 ± 24	0.021
DBP, mmHg	88 ± 12	83 ± 14	0.003
PP, mmHg	45 ± 14	43 ± 15	0.309
MAP, mmHg	103 ± 14	97 ± 16	0.006
cSBP, mmHg	129 ± 18	120 ± 18	< 0.001
**Biochemical variables**			
TC, mmol/L	4.53 ± 1.60	4.52 ± 1.01	0.904
LDL-c, mmol/L	2.79 ± 1.02	2.67 ± 0.89	0.362
TG, mmol/L	1.18 (0.53; 2.85)	1.10 (0.50; 2.40)	0.317
HDL-c, mmol/L	1.25 (0.69; 2.27)	1.32 (0.74; 2.53)	0.250
Glucose, mmol/L	5.34 (3.85; 9.03)	5.18 (4.35; 6.48)	0.529
HbA1c,%	5.92 (5.00; 8.80)	5.46 (4.90; 6.30)	< 0.001
CRP, mg/L	1.11 (0.05; 16.1)	1.38 (0.04; 43.7)	0.072
γ-glutamyltransferase, U/L	23.2 (1.46; 256)	23.4 (0.70; 236)	0.963
**HIV-related parameters**			
CD4 cell count, cell/mm^3^	-	519 ± 263	-
≤ 500 cells/mm^3^, *N* (%)	-	54/106 (50.9)	-
≤ 200 cells/mm^3^, *N* (%)	-	9/106 (8.5)	-
**Renal function**			
SCr, µmol/L	55.9 ± 11.4	57.0 ± 12.8	0.499
CrCl, mL/min	116 (72.0; 208)	97.9 (56.9; 165)	< 0.001
CrCl < 50 mL/min, *N* (%)	0/113	1/113	0.316
eGFR, mL/min/1.73 m^2^	103 (83.2; 123)	103 (74.3; 120)	0.985
eGFR, < 90 mL/min/1.73 m^2^, *N* (%)	14 (12.3)	14 (12.3)	1.000
uACR, mg/mmol	1.43 (0.43; 14.6)	1.89 (0.52; 14.7)	0.720
uACR, 3–30 mg/mmol, *N* (%)	18/102 (17.7)	27/100 (27.0)	0.110
*Health behaviours*			
Self-reported alcohol use, *N* (%)	34/113 (30.1)	35/111 (31.5)	0.815
Self-reported tobacco use, *N* (%)	41/113 (36.3)	43/111 (38.7)	0.704
**Medication use**			
Antihypertensive med, *N* (%)	35 (30.7)	14/109 (12.8)	0.001
Diuretics, *N* (%)	38 (33.3)	20/109 (18.4)	0.010
Statins, *N* (%)	6 (5.3)	1/109 (6.4)	0.063
Anti-inflammatory med, *N* (%)	8 (7.0)	7/109 (6.4)	0.859
Antidiabetic med, *N* (%)	10 (8.8)	0/109 (0.0)	0.002
Anticoagulant med, *N* (%)	9 (7.9)	3/109 (2.8)	0.089
Antiretroviral therapy (ART), *N* (%)	-	85/110 (77.3)	-
≥ 5 years on ART, *N* (%)	-	38/59 (64.4)	-
Metabolic syndrome, *N* (%)	50 (43.9)	32 (28.1)	0.013

s.d., standard deviation; CI, confidence interval; HIV, human immunodeficiency virus; *N*, number of participants; WC, waist circumference; BMI, body mass index; DBP, diastolic blood pressure; SBP, systolic blood pressure; PP, pulse pressure, MAP, mean arterial pressure; cSBP, central systolic blood pressure; TC, Total cholesterol; LDL-cholesterol, low density lipoprotein cholesterol; HDL-cholesterol, high-density lipoprotein- cholesterol; HbA1c%; glycated haemoglobin; CRP, C-reactive protein; SCr, serum creatinine; CrCl, creatinine clearance; eGFR, estimated glomerular filtration rate; uACR, urinary albumin-creatinine ratio; Med, medication; ART, antiretroviral therapy.

Data are arithmetic mean ± s.d. or geometric mean (5th and 95th percentile intervals) for logarithmically transformed variables.

The HIV-infected group had lower WC (*p* < 0.001) and BMI (*p* < 0.001) compared to the HIV-uninfected group. When comparing the cardiovascular measurements, the brachial systolic BP (*p* = 0.021), diastolic BP (*p* = 0.003), cSBP (*p* = 0.006) and mean arterial pressure (*p* < 0.001) were higher in the HIV-uninfected group as compared to the HIV-infected group. With regard to the renal function measurements, the HIV-infected group had lower CrCl (*p* < 0.001), but there was no difference in eGFR and uACR.

After adjusting for WC, diastolic BP, mean arterial pressure and cSBP remained higher in the HIV-uninfected participants (Appendix 1, Table 1), but the brachial systolic BP (*p* = 0.057) and CrCl (*p* = 0.304) no longer differed. When the same analysis was performed for BMI, similar results were observed.

To determine the potential influence of ART, we repeated the comparative analyses between the HIV-uninfected and the HIV-infected groups using ART (*N* = 85) (Appendix 1, Table 2). The results remained the same.

**TABLE 2:** Characteristics of the HIV-uninfected and HIV-infected individuals with the metabolic syndrome.

Variable	HIV-uninfected with MetS *N* = 50	HIV-infected with MetS *N* = 32	*P*
Men, *N* (%)	5 (10.0)	4 (12.5)	0.723
Age, years	53.8 ± 6.5	53.5 ± 6.1	0.844
Urban *N* (%)	19 (38.0)	16 (50.0)	0.283
**Anthropometry**			
WC, cm	102 (85.5; 129)	94.6 (80.0; 120.3)	0.023
BMI, kg/m^2^	32.6 (24.4; 50.0)	27.8 (19.0; 44.0)	0.010
**Cardiovascular measurements**			
SBP, mmHg	139 ± 12	137 ± 29	0.782
DBP, mmHg	91 ± 10	90 ± 14	0.789
PP, mmHg	48 ± 11	47 ± 19	0.839
MAP, mmHg	107 ± 09	106 ± 18	0.772
cSBP, mmHg	133 ± 15	129 ± 20	0.457
**Biochemical variables**			
TC, mmol/L	4.54 ± 1.07	4.66 ± 1.16	0.699
LDL-c, mmol/L	2.88 ± 1.11	2.69 ± 1.00	0.523
TG, mmol/L	1.47 (0.28; 2.72)	1.66 (0.67; 6.70)	0.432
HDL-c, mmol/L	1.02 (0.77; 1.61)	1.34 (0.55; 2.34)	0.316
Glucose, mmol/L	5.71 (3.84; 12.9)	5.56 (4.35; 7.96)	0.684
HbA1c,%	6.28 (5.20; 11.7)	5.71 (5.10; 6.60)	0.040
CRP, mg/L	1.04 (0.05; 15.6)	1.50 (0.04; 29.6)	0.555
γ-glutamyltransferase, U/L	22.7 (2.01; 224)	25.6 (0.70; 325)	0.797
**HIV-related parameters**			
CD4 cell count, cell/mm^3^	-	497 ± 239	-
≤ 500 cells/mm^3^, *N* (%)	-	15/30 (50)	-
≤ 200 cells/mm^3^, *N* (%)	-	2/30 (6.7)	-
**Renal function**			
SCr, µmol/L	55.9 ± 12.2	57.0 ± 12.8	0.694
CrCl, mL/min	133 (86.5; 218)	113 (71.3; 192)	0.050
CrCl < 50 mL/min, *N* (%)	-	-	-
eGFR, mL/min/1.73 m^2^	100 (87.2; 112)	104 (82.5; 129)	0.211
eGFR, < 90 mL/min/1.73 m^2^, *N* (%)	7 (14.0)	3 (9.38)	0.532
uACR, mg/mmol	1.43 (0.49; 20.5)	2.80 (0.47; 25.8)	0.065
uACR, 3–30 mg/mmol, *N* (%)	8/46 (17.4)	13/28 (46.4)	0.007
**Health behaviours**			
Self-reported alcohol use, *N* (%)	9/49 (18.4)	10 (31.3)	0.181
Self-reported tobacco use, *N* (%)	14/49 (28.6)	12 (37.5)	0.400
**Medication use**			
Antihypertensive medication, *N* (%)	22 (44.0)	8 (25.0)	0.081
Diuretics, *N* (%)	24 (48.0)	9 (25.1)	0.073
Statins, *N* (%)	3 (6.0)	0 (0.0)	0.158
Anti-inflammatory medication, *N*(%)	4 (8.0)	2 (6.3)	0.766
Antidiabetic medication, *N* (%)	9 (18)	0 (0.0)	0.011
Anticoagulant medication, *N* (%)	6 (12.0)	2 (6.3)	0.392
Antiretroviral therapy (ART), *N*(%)	-	24 (75.0)	-
≥ 5 years on ART, *N* (%)	-	14/22 (14.0)	-

s.d., standard deviation; CI, confidence interval; HIV, human immunodeficiency virus; *N*, number of participants; WC, waist circumference; BMI, body mass index; DBP, diastolic blood pressure; SBP, systolic blood pressure; PP, pulse pressure, MAP, mean arterial pressure; cSBP, central systolic blood pressure; TC, Total cholesterol; LDL-cholesterol, low density lipoprotein cholesterol; HDL-cholesterol, high-density lipoprotein- cholesterol; HbA1c%, glycated haemoglobin; CRP, C-reactive protein; SCr, serum creatinine; CrCl, creatinine clearance; eGFR, estimated glomerular filtration rate; uACR, urinary albumin-creatinine ratio; Med, medication; ART, antiretroviral therapy.

Data are arithmetic mean ± s.d. or geometric mean (5th and 95th percentile intervals) for logarithmically transformed variables.

We further compared HIV-infected group with MetS and HIV-uninfected group with MetS (Table 2). The BPs did not differ between the groups. However, in the HIV-infected group, the WC (*p* = 0.023), BMI (*p* = 0.010) and CrCl (*p* = 0.050) were lower. However, after adjusting for WC (119 [109; 130] vs. 128 [119; 137]; *p* = 0.213) or BMI (120 [111; 131] vs. 127 [119; 136]; *p* = 0.316), the difference in CrCl disappeared. Furthermore, of this group, a greater proportion had microalbuminuria (46% vs. 17%, *p* = 0.007) compared to the uninfected group, supported by a tendency of higher uACR in the HIV-infected group with MetS (2.80 mg/mmol vs. 1.43 mg/mmol, *p* = 0.065).

**FIGURE 1:**
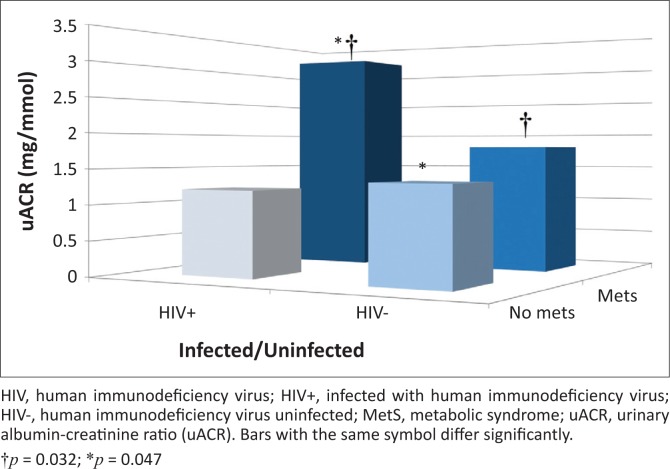
Urinary albumin excretion for HIV-uninfected and HIV-infected individuals with and without metabolic syndrome after adjusting for age, sex and waist circumference.

We also compared uACR between the HIV-infected and HIV-uninfected groups, with and without MetS (Figure 1), while adjusting for age, sex and WC. The mean uACR of the HIV-infected group with MetS (3.16 mg/mmol) was almost double that of the HIV-uninfected group with MetS (1.81 mg/mmol) (*p* = 0.032) despite similar ages and BPs. When comparing the HIV-infected group without MetS to those with MetS, the uACR tended to be lower (1.21 mg/mmol; *p* = 0.11). The uACR was also lower in the HIV-uninfected group without MetS (1.34 mg/mmol), compared to the HIV-infected group with MetS (*p* = 0.047).

We performed multiple regression analysis for renal markers (CrCl, eGFR and uACR) in the total group, HIV-uninfected and HIV-infected groups (Table 3). In the total group, the presence of MetS was positively associated with eGFR (*p* = 0.008).

**TABLE 3:** Multiple regression analysis with markers of renal function as dependent variables.

Variable	CrCl, mL/min			eGFR, mL/min/1.72m^2^			uACR, mg/mmol		
	β	95% CI	*p*	β	95% CI	*p*	β	95% CI	*p*
**HIV uninfected and HIV infected**†									
Age, years	−0.28	−0.37; −0.19	**< 0.001**	−0.44	−0.56; −0.32	**< 0.001**	−0.01	−0.13; 0.12	0.97
Sex, women, men	−0.01	−0.10; 0.09	0.983	0.16	0.04; 0.28	**0.011**	−0.25	−0.38; −0.12	**< 0.001**
cSBP, mmHg	0.06	−0.04; 0.16	0.265	0.01	−0.12; 0.14	0.873	0.36	0.22; 0.50	**< 0.001**
WC, cm	0.63	0.51; 0.75	**< 0.001**	−0.15	−0.31; 0.01	0.053	−0.36	−0.54; 0.19	**< 0.001**
HDL-C, mmol/L	0.01	−0.10; 0.11	0.883	0.05	−0.08; 0.18	0.465	−0.02	−0.17; 0.12	0.754
TG mmol/L	−0.21	−0.31; −0.11	**< 0.001**	−0.11	−0.24; 0.02	0.086	0.11	−0.03; 0.25	0.12
HbA1c, %	−0.02	−0.12; 0.07	0.625	−0.12	−0.24; 0.01	0.079	−0.02	−0.16; 0.12	0.766
CRP, mg/L	0.02	−0.07; 0.12	0.638	−0.03	−0.15; 0.09	0.655	0	−0.13; 0.13	0.975
HIV status, neg/pos	−0.06	−0.15; 0.05	0.279	−0.06	−0.18; 0.07	0.385	0.12	−0.02; 0.29	0.087
MetS, no/yes	0.09	−0.03; 0.23	0.141	0.22	0.06; 0.38	**0.008**	0.11	−0.06; 0.28	0.217
**HIV uninfected**‡									
Age, years	−0.28	−0.41; −0.16	**< 0.001**	−0.45	−0.62; −0.28	**< 0.001**	−0.02	−0.20; 0.15	0.809
Sex, men, women	0.04	−0.09; 0.18	0.522	0.15	−0.03; 0.34	0.109	−0.38	−0.57; −0.19	**< 0.001**
cSBP, mmHg	0.05	−0.08; 0.18	0.471	−0.07	−0.25; 0.11	0.44	0.39	0.21; 0.57	**< 0.001**
WC, cm	0.71	0.56; 0.86	**< 0.001**	−0.13	−0.33; 0.08	0.229	−0.42	−0.63; −0.21	**< 0.001**
HDL-C, mmol/L	−0.05	−0.20; 0.09	0.487	−0.05	−0.25; 0.15	0.622	−0.05	−0.25; 0.15	0.629
TG mmol/L	−0.24	−0.37; −0.11	**< 0.001**	−0.17	−0.34; 0.01	0.065	0.06	−0.13; 0.23	0.622
HbA1c, %	−0.07	−0.20; 0.06	0.319	−0.13	−0.30; 0.05	0.151	0.08	−0.10; 0.26	0.363
CRP, mg/L	−0.02	−0.15; 0.11	0.759	−0.02	−0.19; 0.15	0.804	0.12	−0.06; 0.30	0.193
MetS, no/yes	0.09	−0.08; 0.26	0.288	0.14	−0.09; 0.36	0.228	−0.02	−0.25; 0.21	0.891
**HIV infected**§									
Age, years	−0.31	−0.46; −0.10	**< 0.001**	−0.43	−0.61; −0.26	**< 0.001**	0.03	−0.16; 0.22	0.775
Sex, men, women	−0.02	−0.18; 0.13	0.769	0.15	−0.02; 0.33	0.091	−0.17	−0.36; 0.02	0.086
cSBP, mmHg	0.1	−0.06; 0.27	0.221	0.12	−0.07; 0.31	0.235	0.29	0.08; 0.50	**0.008**
WC, cm	0.5	0.30; 0.69	**< 0.001**	−0.19	−0.41; 0.04	0.106	−0.25	−0.49; −0.11	**0.049**
HDL-C, mmol/L	0.02	−0.15; 0.19	0.813	0.07	−0.13; 0.27	0.498	−0.04	−0.26; 0.17	0.709
TG, mmol/L	−0.21	−0.39; −0.03	**0.023**	−0.09	−0.30; 0.11	0.389	0.13	−0.10; 0.35	0.266
HbA1c, %	0.09	−0.13; 0.22	0.625	−0.08	−0.23; 0.12	0.457	−0.19	−0.40; 0.03	0.099
CRP, mg/L	0.07	−0.09; 0.23	0.37	0.01	−0.18; 0.19	0.951	−0.12	−0.32; 0.08	0.228
MetS, no/yes	0.09	−0.12; 0.30	0.409	0.26	0.02; 0.50	**0.039**	0.26	−0.01; 0.52	0.059
CD4+ count, mm^3^	0.21	0.06; 0.36	**0.009**	0.17	−0.01; 0.35	0.062	−0.13	−0.33; 0.06	0.179
ART, no/ yes	0.07	−0.09; 0.23	0.219	0.13	−0.06; 0.31	0.19	0.07	−0.13; 0.27	0.506

† , HIV-uninfected and HIV-infected (*N* = 228): CrCl, mL/min – *R*^2^ = 0.533; eGFR, mL/min/1.72m2, *R*^2^ = 0.227; uACR, mg/mmol – *R*^2^ = 0.202.

‡ , HIV-uninfected (*N* = 114): CrCl, mL/min – *R*^2^ = 0.583; eGFR, mL/min/1.72m^2^ – *R*^2^ = 0.232; uACR, mg/mmol – *R*^2^ = 0.270.

§ , HIV-infected (*N* = 114): CrCl, mL/min – *R*^2^ = 0.425; eGFR, mL/min/1.72m^2^ – *R*^2^ = 0.231; uACR, mg/mmol – *R*^2^ = 0.152.

Significance of data set in bold.

Total group (HIV-uninfected and HIV-infected, *N* = 228).

β, partial regression coefficient; *R*^2^, adjusted *R*^2^; 95% CI, 95% confidence interval of β; HIV, human immunodeficiency syndrome; N, number of participants; eGFR, estimated glomerular filtration rate; uACR, urinary albumin creatinine ratio; CrCl, creatinine clearance; cSBP, central systolic blood pressure; WC, waist circumference; HDL-C, high-density lipoprotein cholesterol; TG, triglycerides; HbA1c%, glycated haemoglobin; CRP, C-reactive protein; MetS, metabolic syndrome; pos, HIV positive; neg, HIV negative; ART, antiretroviral therapy.

eGFR, uACR, CrCl, cSBP, WC, HDL-C, TG, CRP were logarithmically transformed. Independent variables included in the model include: age, sex, cSBP, WC, HDL-C, TG, HbA1C, CRP, CD^4^ cell count and ART. All independent variables were added at the same time. Bold values indicate *p* ≤ 0.05.

In both the HIV-infected and HIV-uninfected groups, age, WC and TG were associated with CrCl, and age was negatively associated with eGFR. In the HIV-infected group, CD4+ cell count was positively associated with CrCl (*p* = 0.009). The use of ART did not associate with any markers of renal function. When viewing the results for uACR, female sex was associated with increased uACR in the total group (*p* = 0.001) and the HIV-uninfected group (*p* = 0.001), and was borderline in the HIV-infected group (*p* = 0.086). In the total group (*p* < 0.001), the HIV-uninfected group (*p* < 0.001) and the HIV-infected group (*p* = 0.008), uACR was also positively associated with cSBP. We also observed that uACR was negatively associated with WC in the total group (*p* < 0.001), the HIV-uninfected group (*p* < 0.001) and the HIV-infected group (*p* = 0.049). In the HIV-infected group, the presence of MetS was positively associated with eGFR (*p* = 0.039) and had a borderline association with uACR (*p* = 0.059).

### Sensitivity analyses

We repeated the regression analyses and excluded participants not using ART (*N* = 29). The results as presented in Appendix 1, Table 3, remained largely unchanged. For eGFR, the contribution of MetS became weaker (β = 0.21; *p* = 0.15), but for uACR the contribution of MetS became stronger (β = 0.36; *p* = 0.017).

## Discussion

We aimed to determine the prevalence of MetS and to evaluate the renal function in a South African cohort infected with HIV for at least five years. Our main finding was that Africans with HIV infection and MetS had a 43% higher urinary albumin excretion compared to the HIV-uninfected with MetS. However, it is also noteworthy that only 28% of the HIV-infected group had MetS, compared to 44% of their matched, uninfected counterparts.

The latter is in agreement with a cross-sectional study by Jacobson et al.^33^ who reported a lower prevalence of MetS in HIV-infected group as opposed to HIV-uninfected group. We reported a prevalence of 21% in this study population 10 years earlier when they were ART-naïve.^26^ Our current finding of a prevalence of MetS of 28% among the HIV-infected Black people is supported by Julius et al.^8^ who reported a prevalence of 20% among Black South Africans using ART. Hirigo and Tesfaye^34^ reported a similar prevalence of 24% using the IDF critique among Ethiopian HIV-infected individuals on ART. In contrast, other studies have reported a higher prevalence of MetS in HIV-infected individuals, compared to the uninfected.^6,12,35^ In this study, the HIV-infected group had lower obesity and BP measurements, with no differences in lipid and glucose levels, whereas in previous studies the HIV-infected individuals with MetS had a higher prevalence, which was driven by a higher prevalence of impaired metabolic components. The IDF criteria require central obesity as a prerequisite and any other two metabolic components to meet the criteria of MetS.^36^ The lower obesity and BPs in our HIV cohort may explain the lower prevalence of MetS in the HIV-infected participants.

In our study, the majority of HIV-infected participants (77%) were taking ART, which is associated with improved immune status^30^ and either improvement or alteration of MetS components.^6,37^ However, different ART regimens may exert different effects on the metabolic components,^5,6^ which may further explain the lower WC and BP among those on ART in this study. An observational study including HIV-infected participants on ART (with TDF and EFV as part of the regimen) also reported no association between the use of ART and BP among Indians,^38^ while Kaplan et al.^39^ reported a lower prevalence of overweight and hypertension among the HIV-infected individuals, compared to HIV-uninfected individuals. However, when we compared the HIV-infected participants with MetS and HIV-uninfected participants with MetS, no differences were seen in the BP measurements. This indicated the important role of BP in the development of MetS, which may be independent of HIV status.

Without considering the prevalence of MetS, we found that 27% of the HIV-infected individuals had microalbuminuria compared to only 18% of the HIV-uninfected individuals. Szczech et al.^15^ reported a higher prevalence of urinary albumin excretion in an HIV-infected group as compared to uninfected counterparts (11% vs. 2%). Okpa et al.^16^ also reported a prevalence of 15% of microalbuminuria among newly diagnosed HIV-infected Nigerians. However, the latter two studies did not assess MetS.

We aimed to determine whether renal function is affected in those with both HIV and MetS. Microalbuminuria was considerably higher in the HIV-infected individuals with MetS (46%) compared to their uninfected counterparts (17%), despite similar ages and gender distribution. This supports the role of MetS in early renal dysfunction in HIV-infected patients. Our finding of 46% microalbuminuria is higher than a recent report by Pirro et al.,^40^ indicating a prevalence of 17% of microalbuminuria in the HIV-infected with MetS in Italy. However, a control group was not included in the latter study.

Urinary albumin excretion is a well-known marker of renal dysfunction and may precede systematic endothelial dysfunction,^18^ with glomerular permeability to albumin increasing as endothelial dysfunction develops.^41^ Metabolic syndrome is frequently reported in HIV-infected individuals on ART and is associated with both microalbuminuria and endothelial dysfunction.^40^ Furthermore, HIV may directly infect the glomerular epithelial cells, resulting in excretion of albumin.^42^ As tenofovir has nephrotoxic potential,^43^ it mayfurther augment the effect of the HIV infection on the kidneys. Tubular toxicity as a result of tenofovir exposure is frequently reported; however, these reports are controversial.^22^ Glomerular damage may occur as a result of factors such as diabetes, hypertension, MetS and HIV infection which then alter permeability to macromolecules such as albumin and other proteins.^41,42^ In this study, we did not screen the HIV-infected participants for proteinuria, which might have shed some insights into whether the elevated albumin excretion is because of glomerular damage or tubular toxicity. However, in the multivariate analyses, we did not observe an association between the use of ART and the renal function markers in the HIV-infected individuals. Thus, the combination of the MetS, HIV and tenofovir may exacerbate the glomerular permeability, explaining the high albumin excretion in our participants with both MetS and HIV infection.

In the multivariate analyses, renal function was associated with cardiovascular risk factors rather than HIV-associated factors, and ART was not associated with any of the renal function markers. Some studies have reported improvements in renal function with the use of ART and suppressed viral load.^44,45^ In our study, a CD4+ cell count was beneficially associated with CrCl and eGFR, showing that improved immune systems may protect against renal dysfunction.^45^ In addition, during the pre-ART era, the prevalence of microalbuminuria (defined by uACR ≥ 3 mg/mmol - 30 mg/mmol) in HIV-infected individuals was estimated at between 19% and 31%,^19,46^ whereas in the post-ART era, it was estimated at between 8.7% and 11%.^47^

Urinary albumin excretion is an important marker of renal dysfunction and cardiovascular disease risk, even at subclinical levels. Utilisation of uACR may prove beneficial as it is suggested that the substantial renal impairment seen in individuals taking tenofovir is because of pre-existing renal dysfunction, which might be intensified with tenofovir.^48^ It may help to identify HIV-infected individuals with MetS who are potentially at higher risk of renal dysfunction.

## Limitations of the study

This study should be interpreted within the context of its strengths and potential limitations. A limitation of our study is the small sample size of those with MetS. However, the HIV-infected individuals were infected for at least five years and were matched according to age, sex and locality to a control group in order to limit confounders. There were incomplete data on the duration of the ART, but we were ableto determine duration of at least five years, and the participants were on standard first-line triple therapy fixed dose combinations. Tuberculosis testing was not performed for this study; however, information on chronic medication was available. As this was a cross-sectional study, the associations do not indicate cause and effect. This is a well-controlled study, and to our knowledge, this is the first study to investigate the combination of MetS and renal function in an African cohort infected with HIV.

## Conclusion

In conclusion, HIV-infected Africans with MetS had an almost twofold higher urinary albumin excretion compared to the HIV-uninfected controls with MetS. The combination of HIV and MetS indicated an elevated risk for the development of renal disease and cardiovascular disease, and could increase the risk of cardiovascular morbidity and mortality in HIV-infected individuals.

## Appendix 1

**TABLE 1-A1:** Characteristics of HIV-uninfected and HIV-infected individuals after adjusting for waist circumference.

Variable	HIV-uninfected *N* = 114	HIV-infected *N* = 114	*p*
**Cardiovascular measurements**			
SBP, mmHg	133 ± 23.5	127 ± 23.5	0.057
DBP, mmHg	87 ± 13.2	83 ± 13.2	0.029
PP, mmHg	46 ± 14.6	43 ± 14.6	0.278
MAP, mmHg	102 ± 15.9	97 ± 15.9	0.032
cSBP, mmHg	128 ± 18.7	121 ± 18.9	0.004
**Biochemical variables**			
TC, mmol/L	4.49 ± 11.9	4.56 ± 11.9	0.645
LDL-c, mmol/L	2.72 ± 16.4	2.74 ± 16.4	0.911
TG, mmol/L	1.12 (1.02; 1.23)	1.15 (1.05; 1.27)	0.642
HDL-c, mmol/L	1.29 (1.21; 1.38)	1.27 (1.20; 1.36)	0.731
TG/HDL-C ratio	0.87 (0.76; 0.98)	1.10 (0.80; 1.03)	0.603
Glucose, mmol/L	5.23 (5.01; 5.47)	5.22 (4.50; 5.43)	0.937
HbA1c, %	5.84 (5.68; 6.00)	5.53 (5.38; 5.68)	0.007
CRP, mg/L	0.99 (0.67; 1.49)	1.54 (1.03; 2.30)	0.146
**Renal markers**			
CrCl, mL/min	109 (104; 114)	105 (100; 110)	0.304
eGFR, mL/min/1.73 m^2^	102 (101; 106)	103 (100; 105)	0.573
uACR, mg/mmol	1.50 (1.21; 1.87)	1.80 (1.44; 2.24)	0.270

Data are adjusted means ± SD or -95% and +95% CI for logarithmically transformed variables. SD, standard deviation; CI, confidence interval; HIV, human immunodeficiency virus; *N*, number of participants; DBP, diastolic blood pressure; SBP, systolic blood pressure; PP, pulse pressure; MAP, mean arterial pressure; cSBP, central systolic blood pressure; TC, total cholesterol; LDL-cholesterol, low density lipoprotein cholesterol; HDL-cholesterol, high-density lipoprotein cholesterol; TG:HDL-C, triglycerides: High-density lipoprotein cholesterol; CRP, C-reactive protein; CrCl, creatinine clearance; eGFR, estimated glomerular filtration rate; uACR, urinary albumin-creatinine ratio.

**TABLE 2-A1:** Characteristics of the HIV-uninfected and HIV-infected individuals taking antiretroviral therapy.

Variable	HIV-uninfected	HIV-infected taking ART	*p*
**Men**			
*N*	23	17	0.975
%	20.2	20.0	
**Age, years**	53.4 ± 5.5	53.0 ± 5.3	0.636
**Urban *N* (*%*)**			
*N*	46	35	0.906
%	40.4	41.2	
**Anthropometry**			
WC	91.6	82.6	< 0.001
cm	70.5; 123	65.50; 109	
BMI,	27.4	23.0	< 0.001
kg/m^2^	18.0; 44.5	15.6; 35.9	
**Cardiovascular measurements**			
SBP, mmHg	133 ± 21	125 ± 23	0.014
DBP, mmHg	88 ± 12	82 ± 13	0.001
PP, mmHg	45 ± 14	43 ± 15	0.308
MAP, mmHg	103 ± 14	97 ± 15	0.003
cSBP, mmHg	129 ± 18	118 ± 16	< 0.001
**Biochemical variables**			
TC, mmol/L	4.54 ± 1.16	4.50 ± 0.92	0.794
LDL-c, mmol/L	2.79 ± 1.02	2.61 ± 0.87	0.201
TG	1.18	1.14	0.689
mmol/L	0.53; 2.85	0.54; 2.35	
HDL-c	1.25	1.34	0.148
mmol/L	0.69; 2.27	0.79; 2.53	
Glucose, mmol/L	5.34	5.15	0.491
mmol/L	3.85; 9.03	4.26; 6.10	
HbA1c	5.92	5.41	< 0.001
%	5.00; 8.80	4.90; 6.20	
CRP	1.11	1.71	0.160
mg/L	0.05; 16.1	0.04; 44.7	
γ-glutamyltransferase	23.2	26.7	0.538
U/L	1.46; 256	1.24; 225	
**HIV-related parameters**			
CD4+ cell count, cell/mm^3^	-	525 ± 264	-
≤ 500 cells/mm^3^	-		-
*N*	-	37/78	-
%	-	47.4	-
≤ 200 cells/mm^3^	-	-	-
*N*	-	7/78	-
%	-	8.97	-
**Renal function**			
Scr, µmol/L	55.9 ± 11.4	56.4 ± 12.6	0.787
CrCl,	116	100	0.002
mL/min	72.0; 207	56.0; 167	
CrCl, < 50 mL/min			0.245
*N*	0/113	1/84	
%	0.00	1.09	
eGFR,	103	102	0.504
mL/min/1.73 m^2^	83.2; 123	79.2; 120	
eGFR, < 90 mL/min/1.73 m^2^, *N* (%)			0.523
*N*	14	8	
%	12.3	9.41	
uACR	1.43	1.89	0.109
mg/mmol	0.43; 14.6	0.48; 22.6	
uACR, 3 mg/mmol – 30 mg/mmol, *N* (%)			0.232
*N*	18/102	19/76	
%	17.7	25.0	
**Health behaviours**			
Self-reported alcohol use, *N* (%)			0.961
*N*	34/113	25/84	
%	30.1	29.8	
Self-reported tobacco use, *N* (%)			0.667
*N*	41/113	33/84	
%	36.3	39.3	
**Medication use**			
Antihypertensives			0.003
*N*	35	11	
%	30.7	12.9	
Diuretics			0.023
*N*	38	16	
%	33.3	18.8	
Statins			0.122
*N*	6	1	
%	5.3	1.2	
Anti-inflammatories			0.991
*N*	8	6	
%	7.0	7.1	
Antidiabetic agents			0.005
*N*	10	0	
%	8.8	0.0	
Anticoagulants			0.200
*N*	9	3	
%	7.9	3.5	
≥ 5 years on ART			-
*N*	-	38/59	
%	-	64.4	
Metabolic syndrome			0.024
*N*	50	24	
%	43.9	24.2	

Data are arithmetic mean ± s.d. or geometric mean (5th and 95th percentile intervals) for logarithmically transformed variables.

HIV-uninfected, *N* = 114; HIV-infected taking ART, *N* = 85.

s.d., standard deviation; CI, confidence interval; HIV, human immunodeficiency virus; *N*, number of participants; WC, waist circumference; BMI, body mass index; DBP, diastolic blood pressure; SBP, systolic blood pressure; PP, pulse pressure; MAP, mean arterial pressure; cSBP, central systolic blood pressure; TC, total cholesterol; LDL-cholesterol, low density lipoprotein cholesterol; HDL-cholesterol, high-density lipoprotein cholesterol; CRP, C-reactive protein; SCr, serum creatinine; CrCl, creatinine clearance; eGFR, estimated glomerular filtration rate; uACR, urinary albumin-creatinine ratio; Med, medication; ART, antiretroviral therapy; *N*, number.

**TABLE 3-A1:** Multiple regression analysis with markers of renal function as dependent variables, after excluding individuals not using ART.

Variable	CrCl, mL/min			eGFR, mL/min/1.72 m^2^			uACR, mg/mmol		
	β	95% CI	*p*	β	95% CI	*p*	β	95% CI	*p*
Age, years	−0.2	−0.39; 0.58	**0.010**	−0.5	−0.87; 1.09	**< 0.001**	−0.1	0.01; 0.22	0.594
Sex, women, men	−0	0.08; 0.11	0.924	0.24	−0.37; -0.26	**0.027**	−0.1	−0.11; 0.33	0.302
cSBP, mmHg	0.1	−0.09; 0.31	0.337	0.07	−0.01; 0.25	0.568	0.37	−0.61; 0.85	**0.003**
WC, cm	0.5	−0.8; 1.10	**< 0.001**	−0.1	−0.07; 0.35	0.45	−0.3	−0.38; 0.67	0.064
HDL-C, mmol/L	−0	0.05; 0.17	0.768	0.04	0.04; 0.20	0.739	−0	0.08; 0.16	0.875
TG mmol/L	−0.3	−0.38; 0.60	**0.028**	−0.1	−0.05; 0.31	0.461	0.9	−0.05; 0.30	0.479
HbA1c, %	0.02	0.07; 0.14	0.849	−0.1	−0.06; 0.30	0.432	−0.3	−0.40; 0.64	0.872
CRP, mg/L	0.1	−0.09; 0.28	0.329	−0	0.06; 0.15	0.835	−0.2	−0.22; 0.44	0.127
MetS, no/yes	0.05	0.03; 0.23	0.690	0.21	−0.10; 0.39	0.148	0.36	−0.56; 0.86	**0.017**
CD4+ count, mm^3^	0.25	−0.40; 0.59	**0.009**	0.19	−0.26; 0.47	0.079	−0.2	−0.23; 0.45	0.112

CrCl, mL/min, *R*^2^ = 0.533; eGFR, mL/min/1.72 m^2^, *R*^2^ = 0.227; uACR, mg/mmol, *R*^2^ = 0.202.

HIV-infected group using ART (*N* = 85).

β, partial regression coefficient; *R*^2^, adjusted *R*^2^; 95% CI, 95% confidence interval of β; HIV, human immunodeficiency syndrome; eGFR, estimated glomerular filtration rate; uACR, urinaryalbumin creatinine ratio; CrCl, creatinine clearance; cSBP, central systolic blood pressure; WC, waist circumference; HDL-C, high-density lipoprotein cholesterol; TG, triglycerides; HbA1c%, glycosylated haemoglobin; CRP, C-reactive protein; MetS, metabolic syndrome. eGFR, uACR, CrCl, cSBP, WC, HDL-C, TG and CRP were logarithmically transformed. Bold values indicate *p* ≤ 0.05.
